# Fabrication and Assessment of Crumb-Rubber-Modified Coatings with Anticorrosive Properties

**DOI:** 10.3390/ma8010181

**Published:** 2015-01-06

**Authors:** Nasser Al-Aqeeli

**Affiliations:** Mechanical Engineering Department, King Fahd University of Petroleum and Minerals (KFUPM), Dhahran 31261, Saudi Arabia; E-Mail: naqeeli@kfupm.edu.sa; Tel.: +966-13-860-3200; Fax: +966-13-860-3292

**Keywords:** scrap tires, crumb rubber, coatings, erosion, hardness, SEM

## Abstract

Scrap tires continue to be a major source of waste due to the lack of valuable and effective disposal routes. A viable solution to this problem is to recycle crumb rubber (CR)—a granulated material derived from scrap tires—and use it to develop other valuable products. Herein we report the fabrication and characterization of CR-modified coatings with anticorrosive properties on metal substrates. By varying the particle size and concentration of CR, we have determined the coating composition that offers the highest level of erosion protection. Images from a scanning electron microscope (SEM) reveal that CR is homogenously dispersed in the coating, especially when fine particles are used. As the concentration of CR increases, the hardness of the coating decreases as a result of the elastic properties of CR. More importantly, the erosion rate of the coating decreases due to increased ductility. Following Potentiodynamic tests, the utilization of these coatings proved to be beneficial as they showed good protection against aqueous corrosion when tested in 0.5 M NaCl solution. Our newly developed coatings offer an incentive to recycling CR and open up a safe and sustainable route to the disposal of scrap tires.

## 1. Introduction

One of the greatest challenges in solid waste management is the safe disposal of scrap tires. In the United States, consumers throw out more than a quarter of a billion automobile tires every year [1,2]. A large percentage of these tires are sent to landfills where they can contribute to the spread of diseases by becoming breeding grounds for rodents and mosquitos. Some scrap tires are burned using large fires (*i.e.*, pyrolysis), the emissions from which may endanger humans, wildlife and the environment [3]. These methods not only promote the formation of pollutants, but also waste valuable rubber that could have been recycled into new tires for cars, trucks and airplanes. When tires are no longer serviceable, the rubber content is extracted prior to disposal. Waste rubber takes a long time to degrade naturally due to the presence of sulfur cross-links [4]. Incorporating waste rubber into products that have wide appeal and applications is a viable route to reducing scrap tire waste. 

Scrap tires contain three main components: steel belts, fibers and tread rubber. The recycling of scrap tires begins with the mechanical separation of tread rubber from steel belts and fibers, followed by a shredding process that cuts the rubber into small pieces. With the aid of cryogenics, a granulator can further reduce the rubber into fine granules, also known as CR. A large proportion of steel belts and fibers can be recycled and reused, but the same success has not been achieved with CR—due to the difficulty of recasting a thermosetting polymer [5,6,7].

There have been many attempts to use CR as infill materials. One of the major applications is the preparation of concrete for the construction industry. Batayneh *et al.* [8] showed that CR-modified concrete is able to meet weight requirements without sacrificing workability. In addition, they found that CR-modified concrete can absorb a large amount of energy under compressive and flexural loads, has excellent vibration isolation capability and remains intact after failure unlike conventional concrete [9,10,11]. For these reasons, CR-modified concrete is useful in the construction of lightweight walls, building facades and architectural units [11]. A study by Huang *et al.* [12] found that the hardness of CR-modified concrete decreases markedly as CR concentration increases. The finding suggests that CR can only be used as a secondary structural component. Khalilitabas *et al.* [13] assessed the corrosion resistance of steel-reinforced concretes with added CR. They measured the permeability, water absorption, corrosion potential, linear polarization resistance and AC impedance over 150 days of immersion in sodium chloride solution and observed that the interfaces of cement and rubber reduce water permeability. 

CR is also used in the construction of pavements. CR is often employed in jogging tracks, athletic fields and golf courses where it gives surfaces more flexibility and durability [14]. In the United States, manufacturers have established a stable market for CR-modified asphalt—a material that is sprayed onto roads and highways to absorb traffic noise [15,16]. Moreover, CR can be mixed into wall coatings to improve crack resistance and thermal properties [17]. However, the compressive strength of wall coatings is also reduced. 

Tang *et al.* [18] have recently demonstrated the novel use of CR in water purification. They compared the effectiveness of CR-based filters in removing turbidity, particles and plankton from water with that of conventional sand filters. They found that CR filters require less maintenance and experience lower loss of pressure, but are only good enough for secondary treatment (e.g., disinfection). Wang *et al.* [19] have also attempted the fabrication of CR-coated electrodes in microbial fuel cells—an emerging bio-electrochemical technology that has huge economic benefits. They showed that even with two to four layers of CR coatings, the electrode continues to have satisfactory conductivity. 

Herein we report the fabrication of anticorrosive coatings as a new route to recycling CR. Our newly developed coatings [20] showed improved mechanical properties that may benefit the automotive or petrochemical industry. The developed coatings are having 2–3 mm in thickness and can be well suited for the utilization the external surfaces of underground pipes that are used in transporting different fluids. In addition, we have characterized the microstructure of the CR-based coatings and determined the composition that gives the best anticorrosive properties. The enhanced features will increase the incentive to recycle scrap tires. 

## 2. Experimental Section 

### 2.1. CR-Based Coating

**Figure 1:**
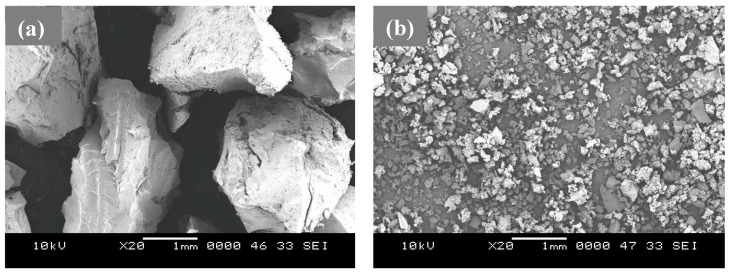
SEM images of crumb rubber (CR) particles. (**a**) Coarse- and (**b**) fine-type CR.

CR was extracted from scrapped tires through a process called shredding, which involves separating the constituents and crushing the rubber into fine granules. CR of two different particle sizes was used in this study: a coarse type measuring 2–3 mm in diameter and a fine type measuring 100–200 μm in diameter. Figure 1 shows pictures of coarse (a) and fine (b) particles. As can be seen from Figure 1, the shape of the coarse CR is not equiaxed and they were able to fit in a 2–3 mm coating. This can be obtained as they have the 3 mm length in 1 dimension and they can be substantially smaller in the other dimensions. The other constituents of the coating are as follows:

- The liquid epoxy resin D.E.R.^TM^ 351, which is produced in the reaction of epichlorohydrin with bisphenol A and bisphenol F;
- The hardener HY 2973, which promotes the hardening of the resin;
- Benzyl alcohol (C_6_H_5_CH_2_OH or BnOH), which acts as the solvent.

Coatings were prepared by adding different amounts of CR, liquid epoxy resin and hardener to 1.5 wt% of benzyl alcohol. CR was added first, and then the mixture was stirred for 1 min. The hardener was added second and the mixture was then stirred for 3 min. Benzyl alcohol was added last, upon which the mixture was stirred until it shows a homogeneous distribution of the coating’s constituents. Metal substrates (*i.e.*, metal pipe surfaces which are carbon steel in these experiments) were coated by manually dipping them into the mixture or by pouring/spraying the mixture on them. It is important to ensure that the surface of substrate is having adequate roughness in order to allow proper adhesion of the coating. Moreover, the coating thickness was maintained at a value of about 2–3 mm in all coatings through controlling the dipping time. The coating was then allowed to dry for 3 h. 

We explored the effect of composition on the mechanical properties of the coating by varying the CR particle size and concentrations of the constituents. We then determined the optimal coating composition that produced the best combination of properties. Table 1 shows the different coating compositions used in our experiment.

**Table 1:** Different compositions of the coatings (% benzyl alcohol is relative to the total weight of the coating). Calculation was based on 50 g of coating.

Exp. No.	Coating compositions
Exp. 1.1	24% coarse CR particles, 76% resin/hardener at 2:1 ratio, 1% benzyl alcohol
Exp. 1.2	35% coarse particles, 65% resin/hardener at 2:1 ratio, 1% benzyl alcohol
Exp. 1.3	50% coarse particles, 50% resin/hardener at 2:1 ratio, 1% benzyl alcohol
Exp. 2.1	24% fine particles, 76% resin/hardener at 2:1 ratio, 2% benzyl alcohol
Exp. 2.2	35% fine particles, 65% resin/hardener at 2:1 ratio, 2% benzyl alcohol
Exp. 2.3	50% fine particles, 50% resin/hardener at 2:1 ratio, 2% benzyl alcohol
Exp. 3.1	24% coarse/fine particles at 1:1 ratio, 76% resin/hardener at 2:1 ratio, 1% benzyl alcohol
Exp. 3.2	35% coarse/fine particles at 1:1 ratio, 65% resin/hardener at 2:1 ratio, 1% benzyl alcohol
Exp. 3.3	50% coarse/fine particles at 1:1 ratio, 50% resin/hardener at 2:1 ratio, 1% benzyl alcohol
Exp. 4.1	24% coarse/fine particles at 4:1 ratio, 76% resin/hardener at 2:1 ratio, 1% benzyl alcohol
Exp. 4.2	35% coarse/fine particles at 4:1 ratio, 65% resin/hardener at 2:1 ratio, 1% benzyl alcohol
Exp. 4.3	50% coarse/fine particles at 4:1 ratio, 50% resin/hardener at 2:1 ratio, 1% benzyl alcohol
Exp. 5.1	24% coarse/fine particles at 1:4 ratio, 76% resin/hardener at 2:1 ratio, 1% benzyl alcohol
Exp. 5.2	35% coarse/fine particles at 1:4 ratio, 65% resin/hardener at 2:1 ratio, 1% benzyl alcohol
Exp. 5.3	50% coarse/fine particles at 1:4 ratio, 50% resin/hardener at 2:1 ratio, 1% benzyl alcohol

### 2.2. Characterization and Testing 

The morphology of the developed coatings was studied using a SEM (JEOL JSM-6460LV 10 keV, Tokyo, Japan). The hardness results were measured using a Brinell hardness tester with a load of 25 kg and an indenter 5 mm in diameter. Ten readings were taken from each sample in multiple locations and the average was recorded. Solid particle erosion tests were conducted at room temperature using a K93700 air jet erosion tester manufactured by KOEHLER Instrument Company, Inc. (New York, NY, USA). Exploratory experiments were conducted to determine the proper erosion parameters in order to properly test the erosion characteristics of the coatings. The erosion testing specifications, including the nozzle diameter and the nozzle length, conform to the ASTM-G76-95 standard [21]. Fifty-micron alumina was used as an erodent at a particle feed rate of 2.5 g/min. The pressure was set to 0.8 bar, the test angle to 90 degrees (for maximum exposure) and the velocity to 60 m/s. The erosion rate is reported as material mass loss per gram of erodent (mg/g). Corrosion tests were carried out in a three-electrode cell, which composed of a specimen as a working electrode, a Pt wire as a counter electrode, and a saturated calomel reference electrode (SCE). The specimens were cut to the size, which can be used in the electrochemical testing flask, ground using 400-grit SiC paper and subsequently washed with distilled water prior to electrochemical tests. The investigations were carried out with an exposed working electrode area of 0.2 cm^2^ in 0.5 M NaCl solution at room temperature in PCI4/750 Gamry potentiostat (Warminster, PA, USA). DC105 corrosion software was used to analyze the Tafel region, while Potentiodynamic polarization experiments were performed at a scan rate of 0.1 mV/s. PD tests were repeated three times to ensure consistency and repeatability of the results.

## 3. Results and Discussion

**Figure 2:**
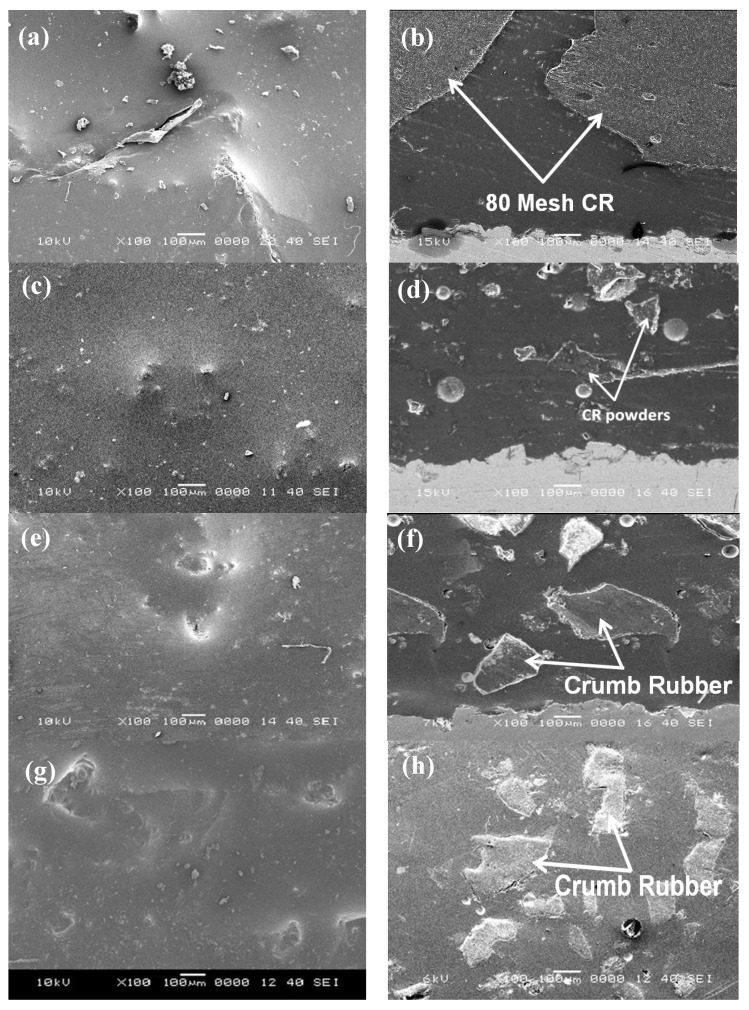

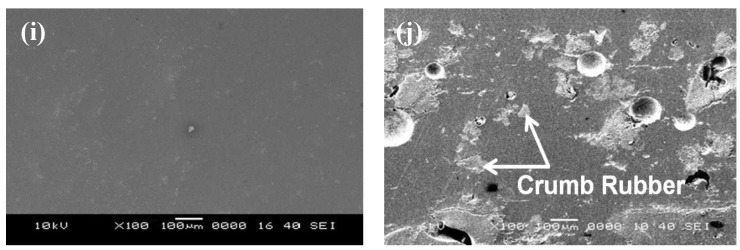
SEM images of the coatings with different compositions of CR: (**a**) top and (**b**) cross-sectional views of a coating made using 35% coarse particles; (**c**) top and (**d**) cross-sectional views of a coating made using 35% fine particles; (**e**) top and (**f**) cross-sectional views of a coating made using 35% coarse/fine particles at 1:1 ratio; (**g**) top and (**h**) cross-sectional views of a coating made using 35% coarse/fine particles at 4:1 ratio; (**i**) top and (**j**) cross-sectional views of a coating made using 35% coarse/fine particles at 1:4 ratio.

In general, the metallic substrates were well covered with CR-modified coatings. We found that a good adhesion requires the CR concentration to be within 24%–50% of the total weight of the coating. Visible voids and non-uniformity develop when the CR concentration is less than 24% or greater than 50%. Figure 2a,b show the top and cross-sectional SEM images of a CR-based coating made using 35% coarse particles. A smooth coating with irregularities was observed. This appears to be due primarily to the larger size of and greater irregularity present in a coarse-particle sample, which makes it difficult for them to mix uniformly with the resin. A cross-sectional view shows the distribution of CR particles from the side, which is essential to the performance of the coating. It is also apparent that the adhesion of the coating to the substrate is good, despite the presence of minimal voids and discontinuities at the interface. Adhesion can be improved further by increasing the surface roughness of the substrate and allowing more entanglement between the coating and the substrate. Figure 2c,d show the top and cross-sectional images of the 35% fine particles. The use of fine particles seems to produce smoother surfaces with no apparent discontinuities. The cross-sectional view also shows the improved dispersion of the CR within the resin, the improved adhesion between the resin and the CR particles and a lack of voids or disengagements. 

Figure 2e,f show the top and cross-sectional SEM images of the CR-modified coating made from a mixture of coarse and fine particles. The total percentage of CR was 35% and the ratio of coarse particles to fine particles was 1:1. The top view does not seem to be very smooth, and there are a few discontinuities on the surface. Nevertheless, the surface coating is improved through the use of fine particles. This could be related to the quality of the mixing that can be achieved when smaller CR particles are added to the coating. However, the cross-sectional view shows the presence of some voids in the coating and some areas of disengagement between the CR particles and the resin. The distribution of the different sizes of CR particles in the resin seems to be adequate while the interface between the coating and substrate has small areas of discontinuities. 

Figure 2g,h show top and cross-sectional SEM images of a coating made using a mixture of coarse and fine particles at the ratio of 4:1. The top view of the SEM image shows irregularities on the surface with no observed voids or discontinuities. The cross-sectional view, however, shows good dispersion of the CR in the coating and the presence of minute voids. The adhesion between the CR and the resin does not seem to be improved as few discontinuities are observed. The interface between the coating and substrate contains a few voids and shows a loss of adhesion. 

Figure 2i,j show top and cross-sectional images of a coating made using a mixture of coarse and fine particles at the ratio of 1:4. On all samples, the resulting surface showed unusual smoothness. It seemed that the additional fine particles might have improved the surface appearance. The cross-sectional views of the SEM images showed the presence of smaller CR particles towards the upper part of the coating while the larger sized CR seems to accumulate toward the lower part of the coating. The images also show more voids and discontinuities, which might be related to poor homogeneity in the mixture of CR and resin. It is apparent that the addition of larger size CR introduces some disturbances into the adhesion and quality of the resulting coating. In summary, the distribution and adhesion of the CR seems to be adequate in all cases and the development of the new coatings seems to be going well. 

**Figure 3:**
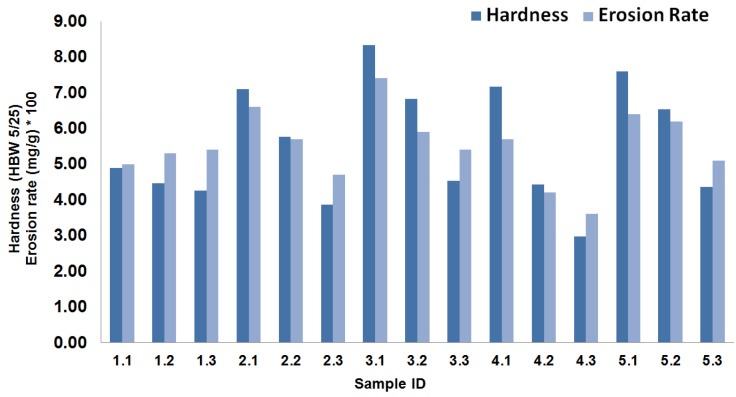
Hardness and erosion rate (multiplied by 100) for all coated samples.

To assess the properties of the newly developed coatings, measurements of the hardness and erosion rate of each material were carried out and presented in Figure 3. The samples are listed by their experiment number and both the hardness and the erosion rates were plotted in the same figure. The erosion rate is multiplied by 100 to enable a side-by-side visual comparison and error bars were removed as there were very small variations in the readings. The concentration of CR in the coatings was 24%, 35% or 50%. The data show an inverse correlation between the amount of CR and hardness. This is due to the fact that CR is relatively soft and increasing its concentration in a coating reduces the bulk hardness of that coating. In some cases, the reduction in hardness is quite substantial, especially in coatings containing coarse and fine particles at 4:1 ratio. In contrast, the coatings with the highest hardness values are those containing coarse and fine particles at 1:1 ratio. By comparison, the hardness of CR-modified coatings containing fine particles only (2.1, 2.2 and 2.3) appears to be greater than those containing coarse particles only (1.1, 1.2 and 1.3). This effect was also observed in 4.1, 4.2 and 4.3 wherein coatings containing 80% coarse particles were used, as well as 5.1, 5.2 and 5.3 where coatings containing 20% coarse particles were used. The results indicate that the hardest coatings are produced by mixing coarse and fine particles in equal parts. 

The erosion rate follows the same trend as the hardness. The coatings exhibiting the highest erosion rates are those with the highest hardness values. These results are comparable with the trends observed by Oka *et al.* [22], where the shape of the erosion curve was found to depend on hardness. In general, as the CR content increases, the erosion rate decreases, which can be explained by the increased amount of soft material. The only group of coatings that does not follow this trend is the group that contains coarse particles only. In this group, as the content of CR increases the erosion rate increases. The erosion rate measurement for this group was repeated multiple times and still showed the same trend. This reversal might be due to the quality of the mixing or to the focused wear by the solid particles on the CR, which can wear out faster than other parts of the coating. The optimal combination of characteristics for a high-quality coating is large hardness and small erosion rate. Unfortunately, this is difficult to achieve, because as we increase the hardness, the material becomes more brittle and cannot accommodate the collision of erodent materials. 

**Figure 4:**
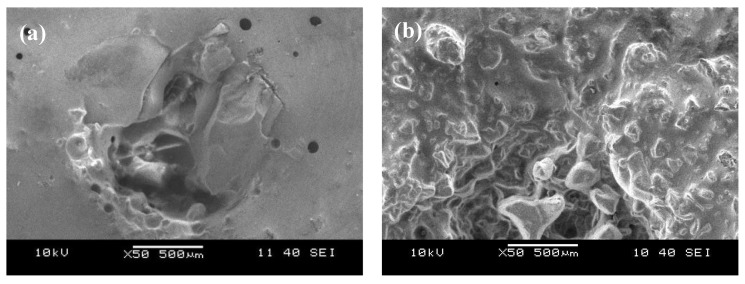
SEM images of eroded surfaces of coatings (**a**) 24% coarse/fine particles at 4:1 ratio and (**b**) 50% fine particles.

The erosion resistance of a material depends largely on the mechanical properties of that material. The work of I. Finnie [23] on pure metals showed that erosion resistance is directly proportional to hardness. For the newly developed coatings presented here, it was found that the erosion rate increases with increasing bulk hardness. To understand the erosion mechanism, SEM images of the eroded surfaces were captured and presented in Figure 4. Those images revealed evidence of material cutting, shredding and localized fractures on the eroded coatings. In general, the erosion mechanism depends on the ductility and brittleness of the material. In ductile materials, the impact of solid particles causes localized plastic deformation and eventually leads to failure. For brittle materials, the impact of solid particles results in cracking and chipping-off of small chunks. 

Ductile erosion can be identified on all the coatings where material was removed by micromechanical deformation and fracture processes. SEM images of representative samples show the trend that was observed in all samples. In such cases, the ductility of a coating increases erosion resistance by absorbing the kinetic energy of impacting particles and plastically deforming the surface while staying within the fracture strain limits [24]. It is also important to mention that the angle of incidence of the impacting particles plays a major role in the erosion rate. It was shown by Hein and Shewmon [25] that at normal incident angles, the ductile materials absorb most of the kinetic energy of incoming particles resulting in lower mass loss. On the other hand, hardness and erosion testing were conducted on neat coating (doesn’t contain CR) to unravel the effect of adding CR into the developed coating. The tested samples were unable to withstand the indentation and started cracking during hardness measurement. In the case of erosion testing, the need for having CR into the coating was more prominent as the neat coating showed very brittle-like fracture and penetration into the coating was more severe. It appears that the addition of CR into the coating provided a foundation for ductile behavior, which was evident by the erosion tracks on the surface of the exposed coatings. 

**Figure 5:**
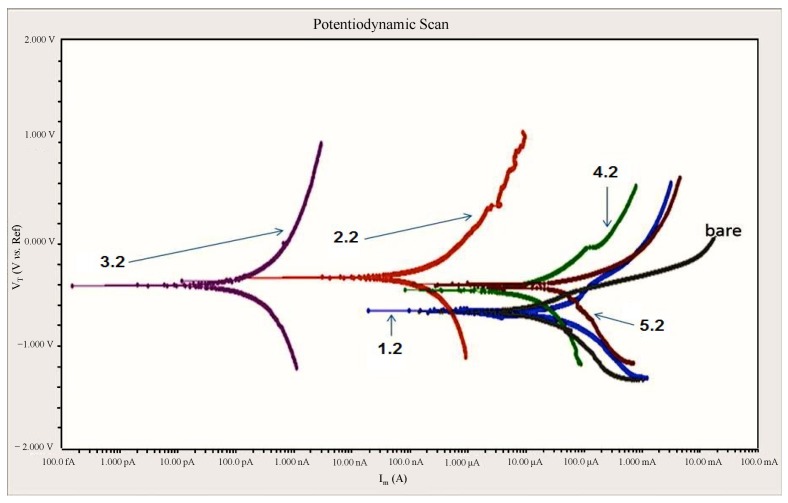
Potentiodynamic Polarization Curves of CR-coated samples and a bare substrate (without coating).

Figure 5 shows the results of Potentiodynamic polarization response of the crumb rubber coated samples. Due to the large number of samples, few samples are presented here since the behaviors of each category of coatings were comparable within the group. All the coated samples showed improved corrosion resistance as compared to the bare steel substrate in terms of corrosion current density, passive current density and corrosion potential. In coated samples, anodic current showed a substantial decrease in current density with an increase in potential, which exhibits the positive effect of coatings on the bare steel surface. Among the coated samples sample 3.3, showed the best results, with the lowest corrosion current density, lowest current density in the passive region and lowest corrosion rate. Corrosion potential of the coated samples was also increased a bit showing a noble character of the coated samples. The order of corrosion resistance of the samples was in the following order: Sample # 3.2 > 2.2 > 4.2 >5.2 >1.2 > bare. It appears that adding the fine and coarse CR particles in equal amounts “1:1” yields the lowest corrosion rate. This is followed by the sample that contained only fine CR particles in the coating. The samples that contained only coarse CR particles had the highest corrosion rate compared to other coated samples. By looking into cross-sectional SEM images and the different structures it seems that the corrosion behavior is primarily dependent on the quality of the coating and the absence of any voids or deformities in the coating. This is evident by the increased corrosion rate of samples 5.2 and 1.2 where the cross-sectional images showed the presence of some voids and areas of discontinuities between the CR particles and the resin. It is important to mention here that since these coatings are polymeric-based they are supposed to be protective against aqueous corrosion since they are non-conductive. However, the observed corrosion rates of these coatings were mainly due to the permeability of the coatings and the low corrosion rate is indicative of good insulation of the substrate to the environment. Testing was also performed on the neat coating (without CR) and the mixture of resin + hardner was not showing any signal during the PD testing. 

## 4. Conclusions 

In this study, we have fabricated anticorrosive coatings using appreciable amounts of CR. These coatings represent a potential new direction in the use and recycling of scrap tires. The addition of fine CR particles produces smoother surfaces, but as the percentage of coarse particles increases, more voids and irregularities develop in the coating surface. As the content of CR increases, the bulk hardness of the coating decreases but the corrosion resistance increases. The result suggests that the ductility of the coating plays a major role in the erosion resistance of the fabricated coating. The corrosion studies showed an improvement in the corrosion resistance of the substrate due to the application of coatings. A substantial decrease in current density was observed when the coating was applied which is an indication of a decreased corrosion rate. Moreover, as the coating was applied the corrosion potential is going towards more positive values, which is indicative of more nobility. We plan to maximize the amount of CR in the coatings, especially for coarse particles, in future studies. Our findings could help reduce the overall cost and increase the competitiveness of CR-modified coatings.
